# Supplementary material for: Somatosensory Auras in Epilepsy: A Narrative Review of the Literature
Source: Medicines (Basel). 2023 Aug 21;10(8):49. doi: 10.3390/medicines10080049 (PMC10456342; doi:10.3390/medicines10080049)
Supplement: Supplementary file 1 [file medicines-10-00049-s001.zip › medicines-2467732-supplementary.pdf]

Table S1. FreeText and MeSH search terms in the US National Library of Medicine.

Category	Search Terms	Results
Epilepsy Somatosensory Auras	("somatosensorial"[All Fields] OR “somatosensory"[All Fields]) AND ("epilepsy"[MeSH Terms] OR "epilepsy"[All Fields] OR "aura"[All Fields]) AND ("epilepsie"[All Fields] OR "epilepsy"[MeSH Terms] OR "epilepsy"[All Fields] OR "epilepsies"[All Fields] OR "epilepsy s"[All Fields])	1671
